# Supplementary material for: Melatonin and MitoEbselen-2 Are Radioprotective Agents to Mitochondria
Source: Genes (Basel). 2022 Dec 23;14(1):45. doi: 10.3390/genes14010045 (PMC9858905; doi:10.3390/genes14010045)
Supplement: Supplementary file 1 [file genes-14-00045-s001.zip › genes-2087961-supplementary.pptx]

## Slide 1
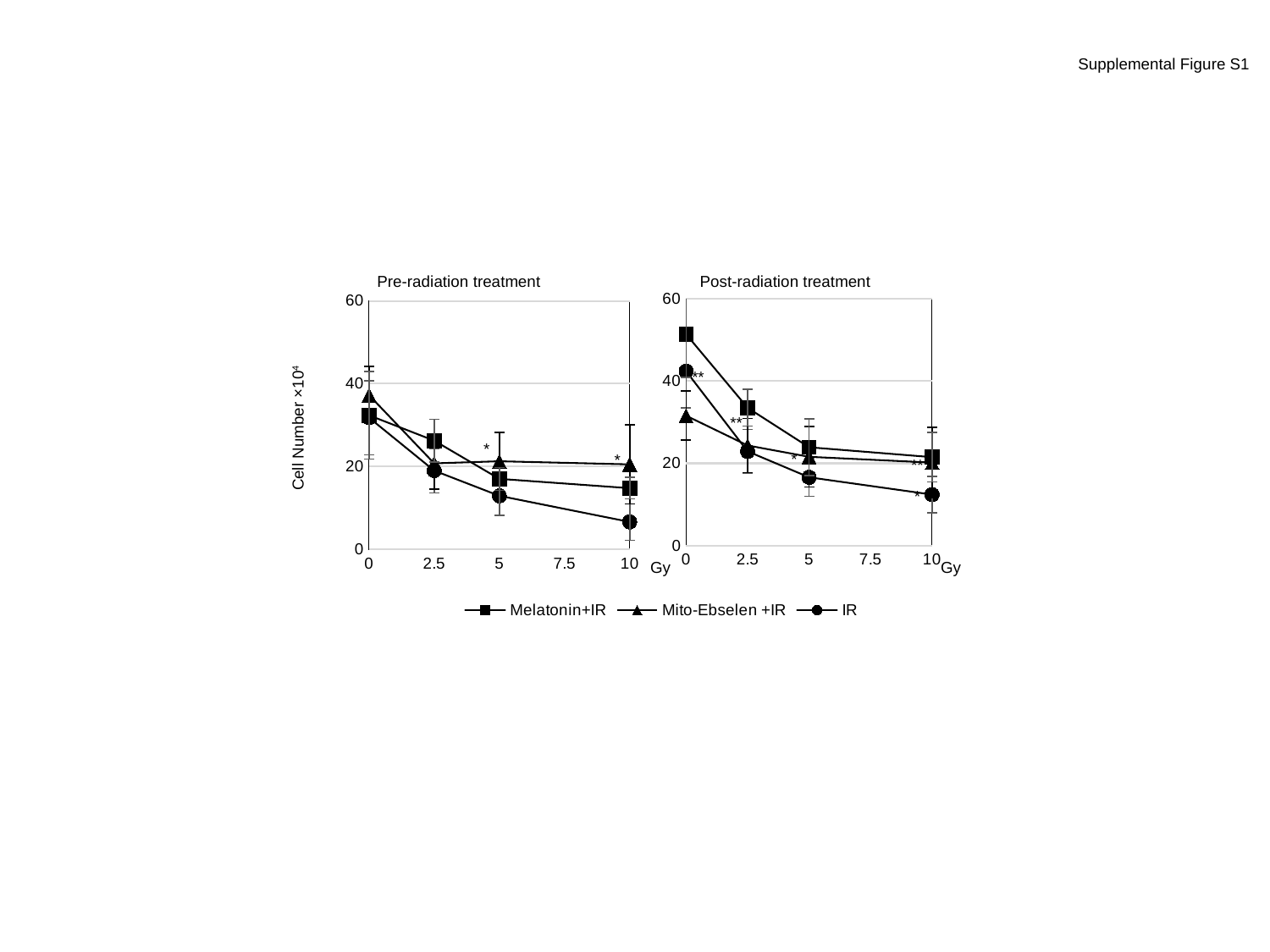

Supplemental Figure S1
Pre-radiation treatment
Post-radiation treatment
### Chart
| Category | | | |
|---|---|---|---|
### Chart
| Category | | | |
|---|---|---|---|**
**
Cell Number ×104
*
*
*
**
*
Gy
Gy
